# Cellular iron homeostasis and metabolism in plant

**DOI:** 10.3389/fpls.2013.00490

**Published:** 2013-12-03

**Authors:** Gianpiero Vigani, Graziano Zocchi, Khurram Bashir, Katrin Philippar, Jean François Briat

**Affiliations:** ^1^Dipartimento di Scienze Agrarie e Ambientali-Produzione, Territorio e Agroenergia, Università degli Studi di MilanoMilano, Italy; ^2^Center for Sustainable Resource Sciences, RIKEN Yokohama campus, RIKENYokohama, Japan; ^3^Department Biology I-Plant Biochemistry and Physiology, Ludwig-Maximilians-University MunichMunich, Germany; ^4^Biochimie and Physiologie Moléculaire des Plantes, Centre National de la Recherche Scientifique, Institut National de la Recherche Agronomique, Université Montpellier 2Montpellier, France

**Keywords:** plant Fe homeostasis, plant metabolism, chloroplast, mitochondrion, vacuole, Fe transporters, Fe-S clusters, plant Fe localization

Iron (Fe) homeostasis represents an important topic in the plant mineral nutrition, since Fe is an essential cofactor for fundamental biochemical activities. Due to the low availability of Fe in most soils, plants are often limited in Fe content. As a consequence, plants must tightly regulate an effective Fe acquisition, distribution, and utilization in root and leaf cells in order to allow for sustainable Fe homeostasis and metabolism. In the past several years, there has been significant progress in understanding how the Fe deficiency responses are regulated and controlled in plants. However, several questions remain still open (Vigani et al., 2013a). Thereby, further work is required in order to fully understand the Fe homeostasis and metabolism in plants.

The aim of this Research Topic is to provide an overview on the new insights of both Fe homeostasis processes and metabolic reprogramming occurring in cellular compartments under different Fe nutritional conditions. Our intent was to recruit a panel of leading experts in the field of plant Fe homeostasis to prepare focused papers advancing our current knowledge in this field and contributing to the development of a comprehensive understanding of iron homeostasis in plants. Within this special issue of Frontiers, 9 original research articles, 4 reviews/mini-reviews and 2 perspective articles are collected.

Four papers are based on “Omic” approaches, which in the past few years provided a huge amount of information about transcriptomic, proteomic, and metabolomic effects induced by Fe deficiency. In particular, Lan et al. (2013) investigated the role of protein phosphorylation in the regulation of cellular Fe homeostasis, by using RNA-seq analysis. The authors revealed networks comprising 87 known or annotated protein kinase and phosphatase genes that could be subdivided into several co-expressed gene modules, assigned to the leucine-rich repeat protein kinase superfamily or associated with biological processes such as “hypotonic salinity response,” “potassium ion import,” and “cellular potassium ion homeostasis.” Furthermore, Rodriguez-Celma et al. (2013), observed a dramatic down-regulation of genes belonging to photosynthesis and tetrapyrrole metabolisms by transcriptomic analysis performed on Fe-deficient Arabidopsis leaves, indicating the presence of a carefully orchestrated balance of potentially toxic tetrapyrrole intermediates and functional end products to avoid photo-oxidative damage. In addition, this study indicated six novel players with putative roles in Fe homeostasis, named Iron Responsive Proteins (IRP) 1–6.

At the proteomic level, the review paper by López-Millán et al. (2013) summarized the new findings that this approach provided during the recent years. Indeed, proteomics has been a powerful tool in the elucidation of general metabolic rearrangements upon Fe deficiency. Fe deficiency has a profound impact on carbon metabolism and on the arrangement of the photosynthetic machinery, with many of these changes conserved amongst plant species. In this context, Khandakar et al. (2013) performed a small-scale proteomic approach on *Hypocampus alba*, confirming the main metabolic strategy of Fe-deficient plants to adjust the metabolism by an economical use of Fe and ATP. The latter including the use of energy to re-programme root morphology and function in response to Fe restriction, which should be a principal strategy for plant roots facing severely suboptimal Fe availability. Additionally they showed also that Fe deficiency affected the alkaloid biosynthesis pathways.

An important topic concerning the nutrient mapping in plant tissues arises in the past recent years. About this issue, two original research papers, dealing with the Fe localization in both tissues and sub-cellular compartments have been published in the present research topic. By using Particle-Induced X-ray Emission (μPIXE) analysis, Schnell Ramos et al. (2013) determined the metal distribution in Arabidopsis seeds. μPIXE induced by a focused ion beam allows multi-elemental mapping in biological samples with high spatial resolution and high sensitivity. This fully quantitative approach revealed important Fe stores outside the endodermal cells. Imaging of imbibed seeds indicates a dynamic localization of metals as Fe and Zn concentrations increase in the subepidermal cell layer of cotyledons after imbibition. Other than quantitative approaches, also qualitative Fe localization techniques have been improved. Indeed, Roschzttardtz et al. (2013) have taken advantage of the Perls/DAB Fe staining procedure to perform a systematic analysis of Fe distribution in roots, leaves, and reproductive organs of the model plant *Arabidopsis thaliana*, using wild-type and mutant genotypes affected in Fe transport and storage. This study has established a reliable atlas of Fe distribution in the Arabidopsis organs, proving and refining long-assumed intracellular locations and uncovering new ones. The authors suggested that the characterization of the “iron map” in Arabidopsis will contribute to identify further actors of Fe movement in plant tissues and cell compartments.

One of the Fe-dependent pathways essential to the cell is the Fe-S cluster assembly. In the present research topic Couturier et al. (2013) reviewed the new actors of this pathways that have been discovered in the past few years, such as glutaredoxin redox enzymes. Further, BolAA and NEET proteins as well as MIP18, MMS19, TAH18, DRE2 are required for the cytosolic machinery of plant Fe-S cluster biogenesis systems.

It is clear that Fe-S clusters biogenesis is strictly related to a correct supply of both Fe and S. In this context Fiorieri et al. (2013) discussed that the interaction between these two nutrients might be of particular importance because Fe starvation influences S nutrition and *vice versa*. As a future perspective they concluded that a co-regulation between Fe and S metabolism exists and that Fe-S cluster availability might be involved in sensing and signaling mechanisms of combined Fe and S deficiencies. Accordingly, also Couturier et al. (2013) suggested the possible involvement of Grx-BolA complexes either in the regulation of Fe-S cluster biogenesis or in the sensing of Fe-S cluster status in organelles where both proteins are simultaneously present.

These findings underline a big open question in the plant Fe homeostasis field: the identification of the cellular Fe sensing and signaling mechanism(s). How such sensing is achieved and how it leads to metabolic adjustments in case of nutrient shortage is mostly unknown. In this research topic, the perspective article by Vigani et al. (2013b) provided a tentative answer, suggesting a possible involvement of some prolyl hydroxylase (P4Hs) belonging to the 2-OG-dioxygenase family proteins in mediating the Fe-induced metabolic adjustment in plants.

Investigating Fe homeostasis at sub-cellular level represents an intriguing topic that provided important discoveries in the past few years. Fe is essential for central organelles such as mitochondria and chloroplasts. Therefore, Fe deficiency responses require wide adjustments of all cellular compartments. Here, papers unraveling mitochondrial as well as vacuolar involvement in Fe homeostasis have been reported. In particular, a timely review by Jain and Connolly (2013) focused on the recent findings related to mitochondrial Fe homeostasis. In particular the authors described the recent identification of both a mitochondrial Fe uptake transporter in rice and a possible role for metal-reductases in Fe uptake by mitochondria. In addition, the authors underlined the recent advances in mitochondrial iron homeostasis with an emphasis on the roles of frataxin and ferritin in Fe trafficking and storage within mitochondria. However, the presence and the role of plant mitochondrial ferritin has been often questioned. In this research topic Vigani et al. (2013c) provided a proof that ferritin is a functional Fe-storage protein in cucumber mitochondria showing that the native 24-mer ferritin complex indeed truly binds Fe(III).

Changes in Fe content in the cell do not affect only Fe-dependent compartments such as mitochondria and chloroplast but also vacuoles. Indeed, the role of the vacuole in Fe-deficient cells is crucial, since the vacuole is involved in homeostasis of both protons and metal concentrations. Dell'Orto et al. (2013) showed the different behavior of cucumber and soybean, developing Fe deficiency responses mainly in relation to the vacuolar proton pump activities. Furthermore, vacuolar sequestration of heavy metals is particularly important during Fe deficiency, when the increased activity of the IRT1 transporter causes excessive uptake of Mn, Ni, and Zn, as well as some other heavy metals. Indeed, Chu et al. (2013) characterized the function and the sub-cellular localization of two new transporters YLS in Arabidopsis plants, demonstrating their localization on tonoplast and endoplasmic reticulum and their involvement in the regulation of homeostasis of heavy metals in the cell. However, these results do not agree with a recently published Plant Cell paper which reported that these YSL transporters (namely YSL4 and YSL6) are chloroplast transporters involved in Fe efflux from this organelle (Divol et al., 2013). These discrepancies between these two studies are so far unexplained and will require additional work to be clarified.

The subcellular analysis of plant Fe homeostasis would benefit from a model organism such as the green algae *Chlamydomonas*. Glaesener et al. (2013) reviewed the progress made on Fe homeostasis in *Chlamydomonas*, detailing the analytical procedures to investigate on it.

Finally, it is important to keep in mind that plants are in interaction with other organisms in their environment, in particular with those living in the soil. Indeed, the complex interactions between plant and soil microbiome make the study on plant Fe nutrition more complex as well as more intriguing. A typical example for this interaction is the legume-rhizobia symbiosis. Brear et al. (2013) underlined the importance of Fe in the legume-rhizobium symbiosis, specifically Fe movement within the symbiotic organ, the nodule. The authors described how the analysis of legume genomes would allow the identification of relevant Fe transporters into the nodule.
